# Preliminary Results of High-Precision Computed Diffusion Weighted Imaging for the Diagnosis of Hepatocellular Carcinoma at 3 Tesla

**DOI:** 10.1097/RCT.0000000000000702

**Published:** 2017-12-29

**Authors:** Motonori Akagi, Yuko Nakamura, Toru Higaki, Yoshiko Matsubara, Hiroaki Terada, Yukiko Honda, Fuminari Tatsugami, Yasutaka Baba, Makoto Iida, Kazuo Awai

**Affiliations:** From the Diagnostic Radiology, Hiroshima University, Hiroshima, Japan.

**Keywords:** diffusion-weighted imaging, computed diffusion-weighted imaging, non-rigid image registration, hepatocellular carcinoma

## Abstract

**Objective:**

To compare the utility of high-precision computed diffusion-weighted imaging (hc-DWI) and conventional computed DWI (cc-DWI) for the diagnosis of hepatocellular carcinoma (HCC) at 3 T.

**Methods:**

We subjected 75 HCC patients to DWI (*b*-value 150 and 600 s/mm^2^). To generate hc-DWI we applied non-rigid image registration to avoid the mis-registration of images obtained with different *b*-values. We defined c-DWI with a *b*-value of 1500 s/mm^2^ using DWI with *b*-value 150 and 600 s/mm^2^ as cc-DWI, and c-DWI with *b*-value 1500 s/mm^2^ using registered DWI with *b*-value 150 and 600 s/mm^2^ as hc-DWI. A radiologist recorded the contrast ratio (CR) between HCC and the surrounding hepatic parenchyma.

**Results:**

The CR for HCC was significantly higher on hc- than cc-DWIs (median 2.0 vs. 1.8, *P* < 0.01).

**Conclusion:**

The CR of HCC can be improved with image registration, indicating that hc-DWI is more useful than cc-DWI for the diagnosis of HCC.

Diffusion-weighted imaging (DWI) is widely used for magnetic resonance imaging (MRI) of the liver. Malignant lesions tend to be more cellular and typically demonstrate impeded diffusion. Consequently, on images obtained with high *b*-values, their signal intensity (SI) is higher than of the background liver parenchyma. Hepatic DWI is useful for the differentiation between metastatic and benign solid hepatic lesions and for estimating the hepatocellular carcinoma (HCC) grade.^1–4^ SI on DWI has been reported to tend to increase as the histologic grade of HCC progressed.^2^ However, DWIs with high *b*-values exhibit a low signal-to-noise ratio (SNR) and such images are severely distorted due to the eddy current elicited by the large diffusion-sensitizing gradients used.

Computed DWI (c-DWI) is a mathematical computation technique that evaluates DWIs acquired with any *b*-value. It uses at least two DWI scans obtained with different *b*-values.^5^ On c-DWI, higher DWI can be simulated based on lower *b*-value images without image quality degradation because c-DWI can suppress the background noise while maintaining the original lesion signal.^5^ Its utility for the detection of malignant lesions such as prostate cancer and hepatic metastases has been reported.^6,7^

Unlike DWI derived from head MRI scans, differences in the organ shape create problems in the generation of hepatic c-DWI^8^ because the apparent diffusion coefficient (ADC) of tissues is calculated from images obtained with two or more *b*-values on a voxel-by-voxel basis. Consequently, the organ shape and location must be conformed on images obtained with different *b*-values. However, respiratory motion can result in deformation and rotation of the liver on images acquired at different *b*-values^9,10^ and the ADC obtained at different *b*-values may not represent the true value. This can result in inaccurate c-DWI findings. Also, as the organ shape may be deformed due to the eddy current on DWI scans with higher *b*-values, the accuracy of c-DWI may be degraded.

Image registration, the process of transforming different images into the same coordinate system, provides an efficient tool to correct misalignments.^11–15^ Image registration methods include rigid, affine, and non-rigid deformable registration.^16^ Unlike rigid and affine registration, non-rigid deformable registration can locally warp the target image to align with the reference image and the non-rigid deformable registration algorithm may correct mis-registration between hepatic DWIs with two *b*-values. We developed high-precision c-DWI (hc-DWI); it corrects mis-registration between DWIs with different *b*-values by applying the non-rigid registration technique. In the current study we compared the clinical utility of hc-DWI and conventional c-DWI (cc-DWI) for the diagnosis of HCC.

## MATERIALS AND METHODS

This retrospective study was approved by our institutional review board; prior informed patient consent was waived because this study was a retrospective observation study. Patient records and information were anonymized and de-identified prior to analysis.

### Study Population

We retrospectively studied 75 HCC patients who underwent hepatic MRI between December 2015 and March 2017. They were 59 males and 16 females; their age ranged from 53 to 89 years (mean 71.7 years). The HCC diagnosis was based on pathologic proof of the tumor burden obtained after partial hepatectomy (n = 19) or imaging findings (n = 56) using (1) typical imaging findings such as obvious enhancement during the hepatic arterial phase with hypo-attenuation compared with the surrounding liver during the equilibrium phase on hepatic CT- or MRI scans in patients at high risk for HCC^17–19^ or (2) the Liver Imaging Reporting And Data Systems (LIRADS) v2017. When tumor was diagnosed LR-4 or 5 using LIRADS v2017, diagnosis of HCC was decided based on the multidisciplinary discussion performed between the radiologists and hepatologists.^20^ The underlying etiology of their chronic liver disease was hepatitis C virus (n = 49), hepatitis B virus (n = 5), alcoholism (n = 8), autoimmune hepatitis (n = 1), and unknown (n = 12).

### MRI Techniques

We used a 3 T scanner (Vantage Titan 3 T, Toshiba Medical Systems, Ohtawara, Japan) to acquire DWIs with a *b*-value of 150 and 600 s/mm^2^. We selected this *b*-value pairing to eliminate the effect of perfusion because its impact on the signal decay at DWI is not negligible at *b*-values below 150–200 s/mm^2.^^21–25^ The scanning parameters for DWI were TR/TE 6666 ms/54 ms, echo train length 40, slice thickness and gap 8 and 2 mm, matrix size 128 × 144, parallel imaging factor 3, receiver bandwidth 1953 Hz/pixel, number of excitations (NEX) 3, *b*-value 150 and 600 s/mm^2^.

We also performed single-breath-hold, fat-suppressed T2-weighted imaging (T2-WI) and dynamic MRI with fat-suppressed T1-weighted (T1W) gradient-echo imaging with 3D acquisition sequences using gadoxetate disodium [EOB-Primovist®, Bayer Yakuhin Ltd., Osaka, Japan; (EOB)]. The parameters for T2-WI were TR/TE 3400 ms/90 ms, echo train length 23, FA 90°, matrix 320 × 192, slice thickness and gap 8 and 2 mm. The acquisition parameters for fat-suppressed T1W gradient-echo imaging with 3D acquisition sequences were section thickness and interval 4 mm, TR/TE 3.0 ms/1.1 ms, FA 12°, field-of-view 36 cm, matrix 288 × 192, parallel imaging factor 2, acquisition time 18 s.

After pre-enhanced scanning, we injected EOB intravenously and acquired four-phase EOB-enhanced scans of the liver during the arterial and portal venous phase (AP, PVP), the transitional, and the hepatobiliary phase (HBP). The scan timing for AP was determined by test injection of 0.5 mL EOB. Scanning during AP was at the aortic transit time calculated from test injection images plus 7 s after the start of the EOB injection. Scanning during PVP and HBP was at 1 and 20 min after the start of the EOB injection. We defined the transitional phase as the 180 s after the start of EOB injection.

We administered EOB at a dose of 25 μmol/kg and at a rate of 2.0 mL/s; flushing was with 20 mL saline using a power injector (Sonic Shot 50; Nemoto-Kyorindo, Tokyo, Japan).

### Generation of Conventional c-DWI

As described elsewhere,^5^ we calculated conventional c-DWI (cc-DWI) by using DWIs with a *b*-value of 150 and 600 s/mm^2^. Briefly, first the ADC was calculated as *ADC* = ln[−*S*_600_/*S*_150_]/(*b*_600_ − *b*_150_), using two measured DWI signals where *S*_600_ and *S*_150_ are the signal intensity at *b* = 600 and *b* = 150 s/mm^2^, respectively, based on a mono-exponential model. ADC maps were constructed using this equation and voxel-wise calculation. Then the c-DWI signal at *b* = *b*_*c*_ was obtained with the equation *S*_*c*_ = *S*_0_ × exp[−(*b*_*c*_ − *b*_0_)*ADC*].

We generated c-DWIs using a software program (computed DWI; Toshiba Medical Systems, Ohtawara, Japan).^5,6^ The time required for calculating c-DWI was less than 1 min.

### Generation of High-precision c-DWI (Fig. 1)

To generate high-precision c-DWIs (hc-DWIs), we preprocessed 150 and 600 s/mm^2^ DWIs before calculating the ADC maps. We registered DWIs with a *b*-value of 150 and 600 s/mm^2^ onto T1WIs during PVP using a non-rigid deformable registration technique. We used T1WIs obtained during PVP as reference images because they contain relatively few artifacts, provide a good SNR and because the contrast is similar to DWIs with well-preserved vessel-liver contrast. We applied a B-spline-based deformable image registration algorithm with mutual information using 3D Slicer software.^26,27^ Then we calculated hc-DWIs with a *b*-value of 1500 s/mm^2^ using the preprocessed DWIs and applied the same method as for cc-DWI.

**FIGURE 1:**
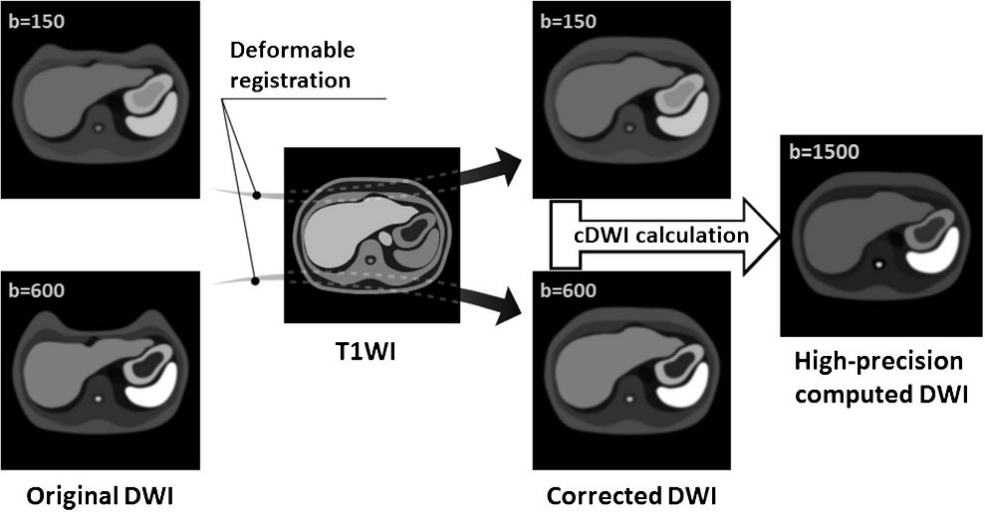
Image processing for the generation of hc-DWIs. We pre-processed DWIs with a *b*-value of 150 and 600 s/mm^2^ before calculating the ADC maps. We registered these DWIs onto T1WIs acquired during PVP using a non-rigid deformable registration technique and then calculated hc-DWIs with a *b*-value of 1500 s/mm^2^ using the preprocessed DWIs.

### Image Evaluation

#### Reference Standards

One board-certified radiologist with 14 years of experience confirmed the HCC site using typical imaging findings such as obvious enhancement during AP with hypo-intensity during HBP on EOB-enhanced MRI scans or LIRADS v2017.^17–20,28^ The reader presented the HCC site to another radiologist (5 years of experience) who then evaluated the signal intensity of the HCC on cc- and hc-DWIs.

#### Quantitative Evaluation

**FIGURE 2:**
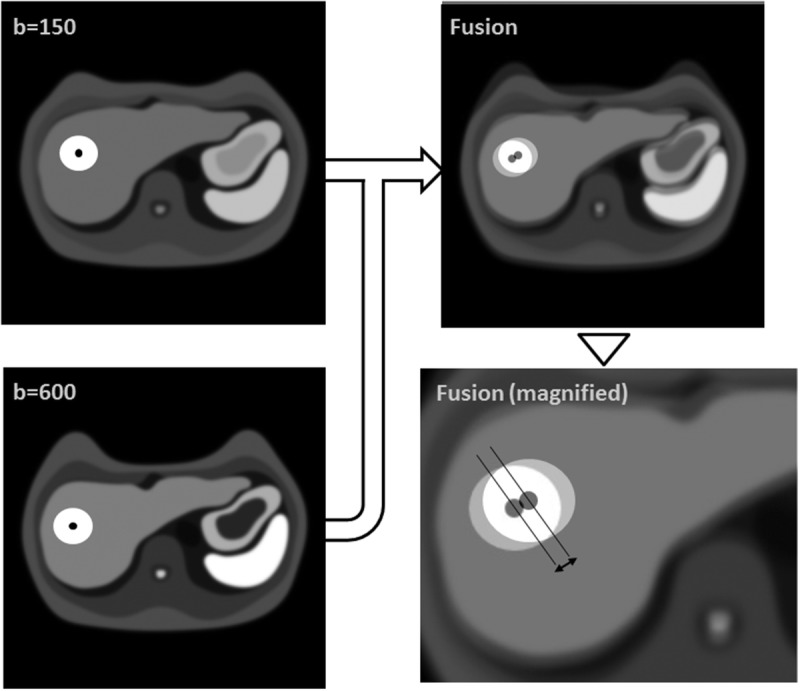
Calculation of the distance between the tumor gravity center on *b*-value of 150 and 600 s/mm^2^ DWIs. White circle indicate the tumor and black dot is located on the tumor gravity center. DWI with *b*-value of 150 s/mm^2^ was fused onto DWI with *b*-value of 600 s/mm^2^ (fusion image). Fusion image indicated the tumor location on *b*-value of 150 s/mm^2^ DWI was shifted from that on *b*-value of 600 s/mm^2^ DWI without registration. On the fusion image (magnified) the arrow indicates the distance between the tumor gravity center on *b*-value of 150 and 600 s/mm^2^ DWIs. The distance should be decreased if registration corrected mis-registration between the two DWIs.

The tumor location including tumor gravity center on *b*-value of 150 and 600 s/mm^2^ DWIs should be identical if registration corrected mis-registration between the two DWIs. The second reader placed regions of interest (ROIs) encompassing whole tumor manually. High intensity region as tumor area was segmented in the ROI using Otsu’s method.^29,30^ This algorithm assumes that the image contains two classes of pixels following a bi-modal histogram. It then calculates the optimum threshold separating the two classes. We defined a gravity center calculating from tumor area using ImageJ (http://rsb.info.nih.gov/ij/) with a plugin (in house) automatically as tumor gravity center. The reader calculated the distance between the gravity center of the tumor on 150 and 600 s/mm^2^ DWIs before and after registration (Fig. 2). The reader also placed ROIs on the HCC and the surrounding hepatic parenchyma on cc-DWI and hc-DWI. The ROIs encompassed the entire tumor. An ROI of at least 1.0 cm^2^ was placed in the surrounding hepatic parenchyma at the level of the hepatic hilum; vascular structures and hepatic space-occupying lesions were avoided. The contrast ratio (CR) between the HCC and the surrounding hepatic parenchyma on each DWI was then calculated using the equation: CR = SI of the HCC/SI of the surrounding hepatic parenchyma.^6^

### Statistical Analysis

For quantitative analysis we recorded statistical differences before and after registration in the distance between the tumor gravity center on 150 and 600 s/mm^2^ DWIs and the CR of each DWI using the two-sided Wilcoxon signed-rank test. We also performed subset analysis based on the tumor size using 20-mm thresholds.

All statistical analyses were performed using free statistical software (R version 2.15.0). Differences of *P* < 0.05 were considered statistically significant.

## RESULTS

The median HCC diameter was 17.0 mm (range 7.0–130.0 mm). Of the 75 HCCs 43 were smaller and 32 were larger than 20 mm.

**FIGURE 3:**
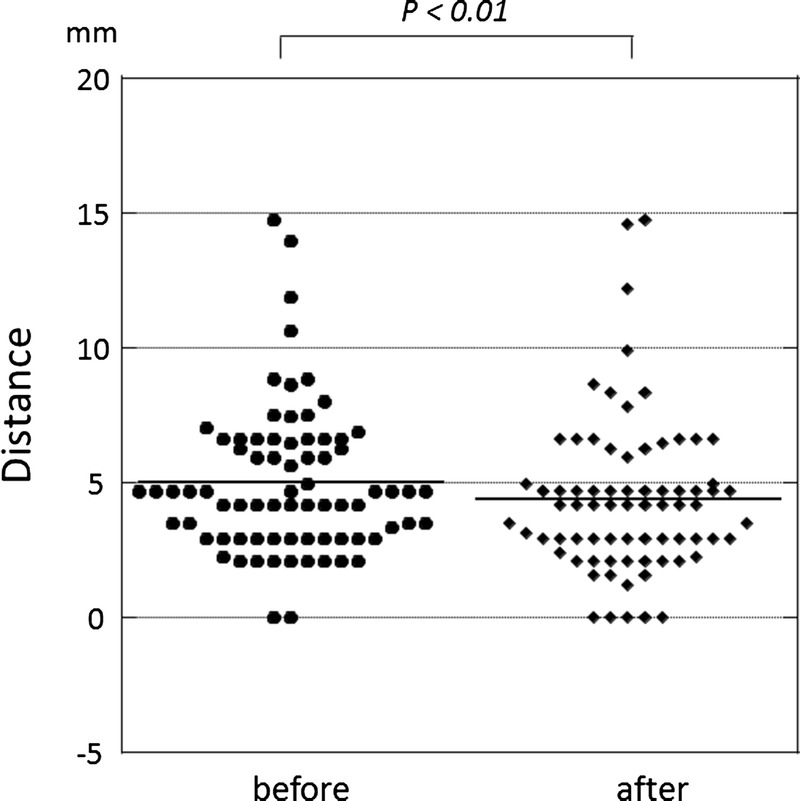
Distance between the tumor gravity center on 150 and 600 s/mm^2^ DWIs before and after registration. It is significantly lower on after- than before registration (*P* < 0.01).

**FIGURE 4:**
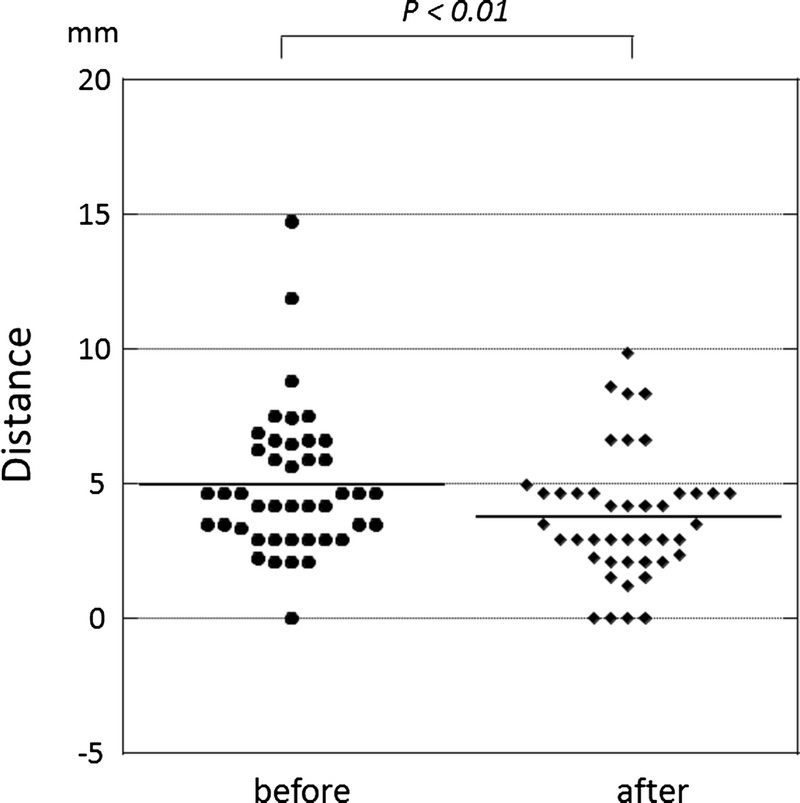
Distance between the tumor gravity center on 150 and 600 s/mm^2^ DWIs before and after registration for HCCs smaller than 20 mm. It is significantly lower after- than before registration (*P* < 0.01).

**FIGURE 5:**
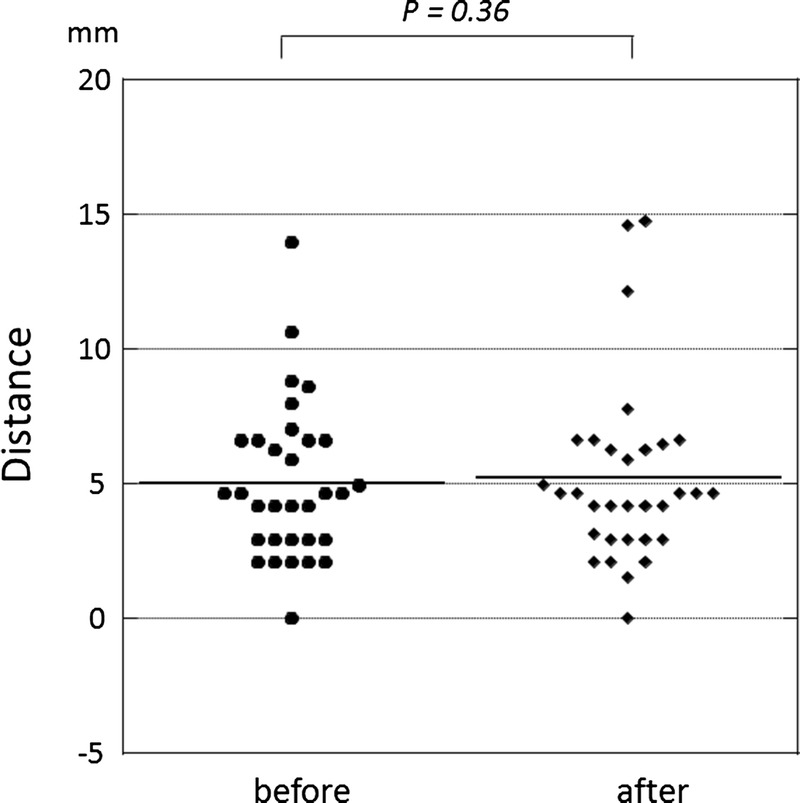
Distance between the tumor gravity center on 150 and 600 s/mm^2^ DWIs before and after registration for HCCs larger than 20 mm. The difference is not significant (*P* = 0.36).

The distance between the tumor gravity center on DWIs with *b*-value 150 and 600 s/mm^2^ before and after registration was 4.7 mm (range 0.0–14.7 mm), and 4.2 mm (range 0.0–14.7 mm), respectively; it was significantly lower after registration than that before registration (*P* < 0.01) (Fig. 3). The results of our subset quantitative analysis based on the HCC size are shown in Figures 4 and 5. For HCCs smaller than 20 mm the median distance before and after registration was 4.7 mm (range 0.0–14.7 mm), and 3.5 mm (range 0.0–9.9 mm), respectively; distance after registration was lower compared to that before registration with significant difference (*P* < 0.01) (Fig. 4). For HCCs larger than 20 mm these measurements were 4.7 mm (range 0.0–14.0 mm) and 4.7 mm (range 0.0–14.7 mm), respectively; they were not significantly different (*P* = 0.36) (Fig. 5).

**FIGURE 6:**
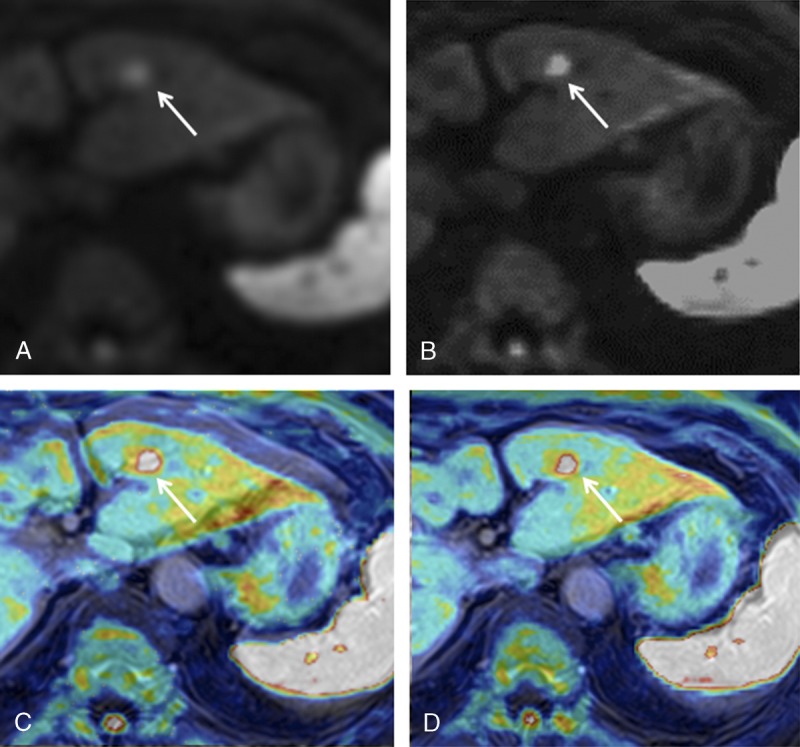
HCC in an 82-year-old woman. A, cc-DWI; B, hc-DWI; C, cc-DWI (color image) fused onto the T1WI obtained during PVP (grayscale image); D, hc-DWI (color image) fused onto the T1WI acquired during PVP (grayscale image). The HCC (arrow) is more clearly depicted on the hc- than the cc-DWI. Note displacement of the liver surface on the cc-DWI and the T1WI scan. The mis-registration on the cc-is corrected on the hc-DWI. Figure 6 can be viewed online in color at www.jcat.org.

**FIGURE 7:**
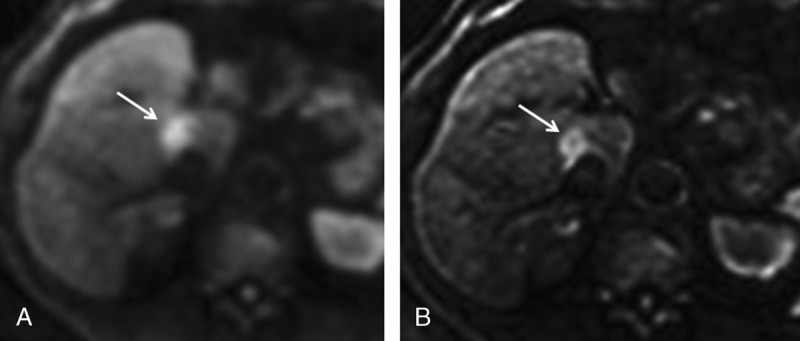
HCC in a 69-year-old man. A, cc-DWI; B, hc-DWI. The HCC (arrow) is more clearly visualized on the hc- than the cc-DWI.

**FIGURE 8:**
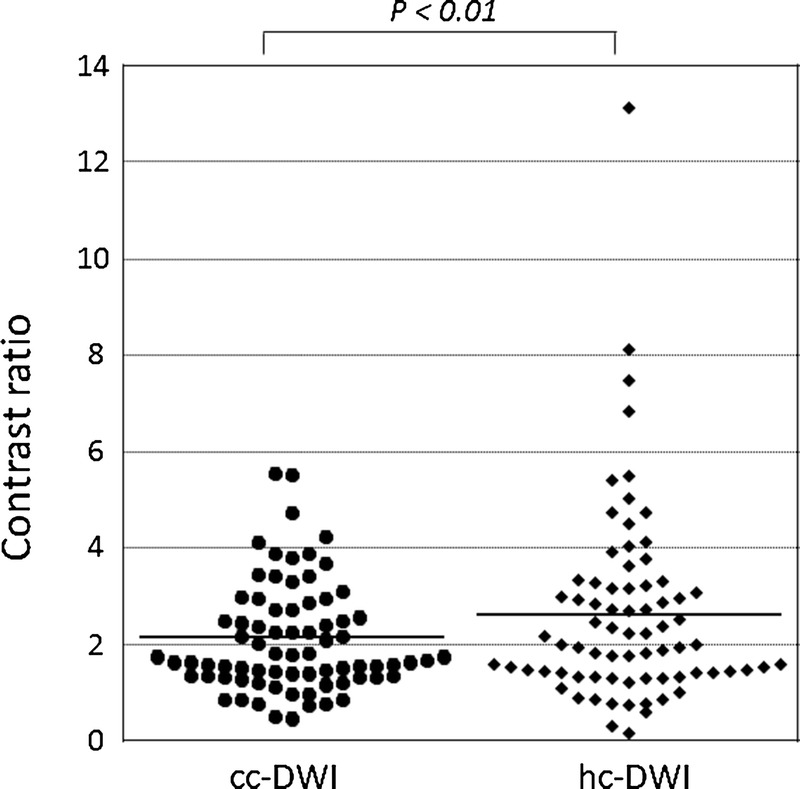
CR of HCCs on cc-DWI and hc-DWI. The solid line indicates the mean CR value in each group. It is higher on hc- than on cc-DWI (*P* < 0.01).

**FIGURE 9:**
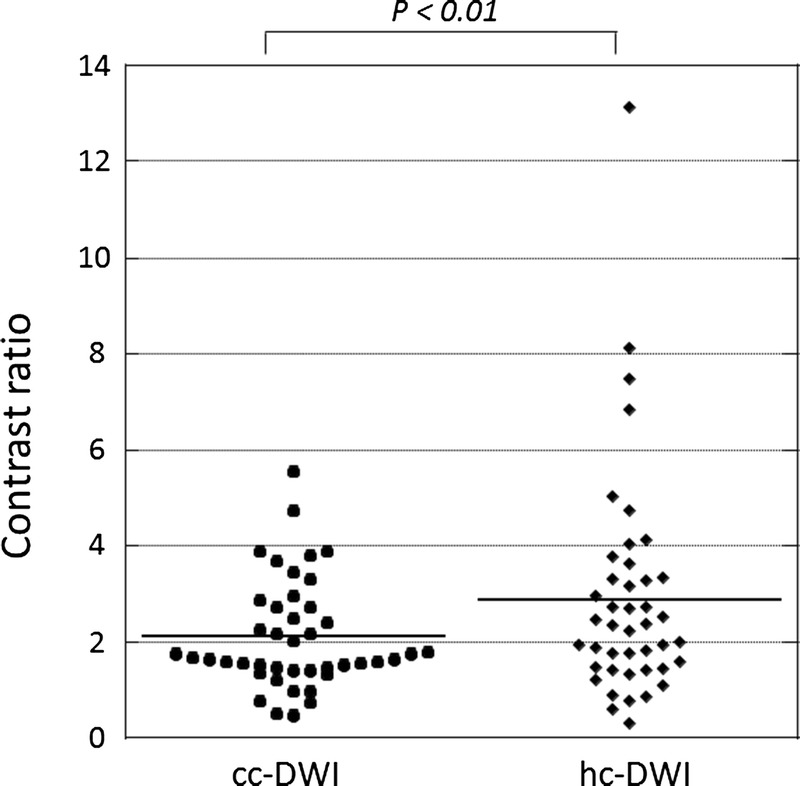
CR of HCCs smaller than 20 mm on cc-DWI and hc-DWI. The solid line indicates the mean CR value in each group. It is significantly higher on hc- than cc-DWI (*P* < 0.01).

**FIGURE 10:**
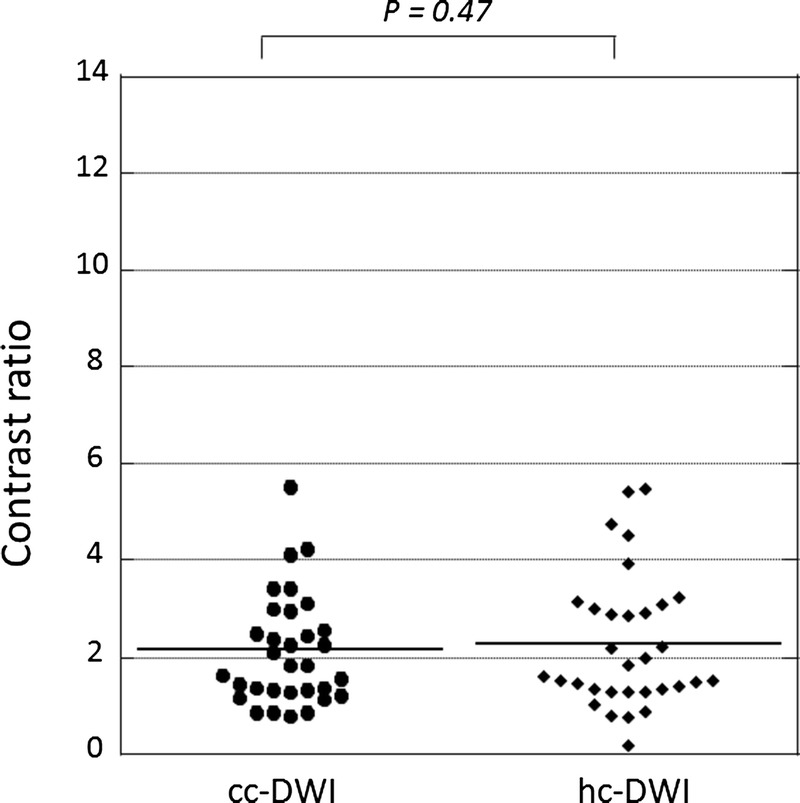
CR of HCCs larger than 20 mm on cc-DWI and hc-DWI. The solid line indicates the mean CR value in each group. There is no significant difference (*P* = 0.47).

Representative images are shown in Figures 6 and 7. The median CR on cc-DWI and hc-DWI was 1.8 (range 0.5–5.6), and 2.0 (range 0.2–13.1), respectively; it was significantly higher on hc-DWI than cc-DWI (*P* < 0.01) (Fig. 8). The results of subset quantitative analysis based on the HCC size are shown in Figures 9 and 10. For HCCs smaller than 20 mm the median CR on cc-DWI and hc-DWI was 1.7 (range 0.5–5.6) and 2.3 (range 0.3–13.1), respectively; the CR on hc-DWI was higher compared to that on cc-DWI with significant difference (*P* < 0.01) (Fig. 9); for HCCs larger than 20 mm these values were 1.8 (range 0.8–5.5) and 1.7 (range 0.2–5.5) and not significantly different (*P* = 0.47) (Fig. 10).

## DISCUSSION

We found that the distance between the HCC gravity center on DWIs with a *b*-value of 150 and 600 s/mm^2^ was significantly lower after than before registration and that the CR was significantly higher on hc- than cc-DWIs. The accurate diagnosis of malignant tumors including HCCs requires a large CR. The increasing availability of 3 T systems makes DWI optimization possible. However, despite the potential advantages of imaging at a higher field strength,^31^ the image quality of 3 T DWIs has been reported to be worse than of 1.5 T images.^32–34^ Thus, we thought that hc-DWI was useful especially at 3 T because hc-DWI can yield better image quality of DWI rather than cc-DWI.

Subset analysis for HCCs smaller than 20 mm addressing the distance between the gravity center of HCCs on DWIs with a *b*-value of 150 and 600 s/mm^2^ revealed that it was significantly lower after- than before registration. This was not the case in tumors larger than 20 mm. Also, the CR was significantly higher on hc- than cc-DWIs for HCCs smaller than 20 mm. For tumors larger than 20 mm the CR on cc- and hc-DWIs was not significantly different. These findings indicate that mis-registration had a greater effect on small- than larger HCCs. Small HCCs commonly exhibit atypical enhancement patterns on dynamic images and the incidence of arterial hypervascularity is lower.^35,36^ Therefore, additional advances in imaging technology are needed to improve the diagnosis of small HCCs.^37,38^ Based on our observations we think that hc-DWI, which yields a larger CR than cc-DWI especially for small HCCs, is the superior imaging method.

DWI yields the lowest SNR among various sequences and DWIs are severely distorted due to the eddy current. Therefore, DWIs should be registered to images of another sequence with a better SNR and contrast similar to DWI scans. T1WI performed during PVP fulfill these requirements and the vessel-liver contrast is well preserved. Thus, we registered DWIs to T1WI during PVP.

c-DWI is based on a mono- rather than a bi-exponential model. As the former does not consider the effect of perfusion, conventional c-DWI results may be affected not only by true molecular- but also by perfusion-related diffusion.^5,6,21,22^ Earlier studies showed that the assessment of true diffusion was diagnostically superior to ADC evaluation for the differentiation between benign and malignant lesions.^39,40^ Therefore we selected *b*-value pairing (150 and 600 s/mm^2^) to eliminate the effect of perfusion because its impact on signal decay on DWIs is not negligible at *b*-values below 150–200 s/mm^2.^^21–25^ Thus, although c-DWI is based on a mono-exponential model, our findings may reflect only the effect of diffusion and additional studies are needed to verify the utility of the hc-DWI technique we employed on this point.

Our study has some limitations. The study population was relatively small, the nature of our investigation was retrospective and it was carried out at a single institution. Moreover, we included HCCs regardless of size although large tumors tend to have necrosis within the tumor unlike small tumors. CR may be changed due to the presence of necrosis, indicating that further study is needed with consideration whether necrosis is present or not inside of the tumor. Therefore we consider our findings to be preliminary. We did not compare hc-DWIs with the original DWIs acquired with a *b*-value of 1500 s/mm^2^. Actually, the original DWIs with high *b*-values including 1500 s/mm^2^ exhibit a low SNR and such images are severely distorted due to the eddy current elicited by the large diffusion-sensitizing gradients used. Thus, it is required to increase the number of NEX for improvement of SNR. However, doubling the NEX only improves the SNR by the square root of two although scanning time doubles, indicating that scan time should be increased longer than double time for doubling SNR and such sequence with long time scan is difficult to perform in clinical setting. In addition, TE may be increased on DWI with high *b*-value due to limitation of the scanner, meaning that T2 shine-through effect may increase.^41,42^ Therefore, the original DWI with *b*-value of 1500 s/mm^2^ was not performed. Moreover, the diagnostic superiority of c-DWI without registration over original DWI for the detection of malignant lesion such as prostate cancer and hepatic metastases has been reported.^6,7,43^ Also, we created c-DWIs with *b*-values of 1500 s/mm^2^ only, although much higher *b*-values may yield better results. However, higher *b*-value images feature a poor SNR and more studies are needed to identify the optimal *b*-value for the diagnosis of HCCs. We performed DWI after the delivery of gadoxetate disodium and its uptake may have affected the SI on DWIs. However, we think that uptake in the hepatic parenchyma had a negligible effect on the diffusion SI because the value of parameters related to diffusion weighting were not significantly different before and after gadoxetate disodium administration.^44^ Lastly, we only evaluated HCCs although DWI play a supportive role in the diagnosis of HCC.^20^ In addition, we have not evaluated other hepatic lesions, meaning that it may be difficult to conclude that hc-DWI is useful for establishing the diagnosis of HCC only with our results. Additional studies are needed to verify the utility of hc-DWI for differentiating benign from malignant lesions, for diagnosing hepatic tumors, and for determining the HCC grade.

In conclusion, the distance between the HCC gravity center on DWI with *b*-value 150 and 600 s/mm^2^ after registration was significantly lower than before registration and the CR was higher on hc- than cc-DWIs, especially for HCCs smaller than 20 mm. Taken together, our findings suggest that hc- is superior to cc-DWI for the diagnosis of HCC.
